# The journey of Europeans with musculoskeletal complaints: the creation of the SPIDeRR’s personas

**DOI:** 10.1016/j.ero.2026.100177

**Published:** 2026-05-05

**Authors:** Teresa Otón, Felix Muehlensiepen, Rachel Knevel, María José Villalobos-Quesada, Barbara Bislawska Axnäs, Harriet Morf, Karin Stratingh, Marina Pérez, Estíbaliz Loza, Loreto Carmona

**Affiliations:** 1 Instituto de Salud Musculoesquelética, Madrid, Spain; 2 Centre for Health Services Research, Faculty of Health Sciences Brandenburg, Brandenburg Medical School Theodor Fontane, Rüdersdorf bei Berlin, Germany; 3 Deutsches Herzzentrum der Charité, Department of Cardiology, Angiology and Intensive Care Medicine, Berlin, Germany; 4 Department of Rheumatology, Leiden University Medical Centre, Leiden, Netherlands; 5 Department of Rheumatology, Newcastle University Translational and Clinical Research Institute, Newcastle upon Tyne, UK; 6 Pattern Recognition Group, University of Technology Delft, Delft, Netherlands; 7 National eHealth Living Lab, Leiden University Medical Centre, Leiden, Netherlands; 8 Elsa Science, Stockholm, Sweden; 9 Department of Medicine 3—Rheumatology & Immunology, Friedrich-Alexander-Universität (FAU) Erlangen-Nürnberg and Uniklinikum Erlangen, Erlangen, Germany; 10 Deutsches Zentrum für Immuntherapie (DZI), Friedrich-Alexander-Universität Erlangen-Nürnberg and Uniklinikum Erlangen, Erlangen, Germany; 11 Dutch Arthritis Society, Amsterdam, Netherlands; 12 Pontia Design, Berlin, Germany

## Abstract

**Objectives:**

The Stratification of Patients Using Advanced Integrative Modelling of Data Routinely Acquired for Diagnosing Rheumatic Complaints (SPIDeRR) project aims to improve the patient journey for individuals with musculoskeletal (MSK) complaints due to rheumatic and musculoskeletal diseases (RMDs). The primary objective was to understand patients’ experiences navigating the healthcare system to achieve timely diagnosis and treatment for RMDs. Secondary objectives included identifying challenges and opportunities for implementing digital tools for help seeking and diagnosis, thus enhancing access to appropriate treatments.

**Methods:**

Based on the patient experience mapping framework, 3 substudies were conducted as follows: (i) a systematic review and stakeholder surveys to identify key stages and touchpoints; (ii) focus groups to gather additional insights; and (iii) synthesis of findings into visual maps and personas highlighting gaps and potential solutions.

**Results:**

Substudy 1 (36 studies and 247 survey participants) revealed that navigating the healthcare systems in Europe is complex, facing significant barriers, such as limited specialist access, knowledge gaps, and inconsistent treatment pathways for people with RMDs. Substudy 2 (28 participants with and without RMDs) found that initial symptom management may delay proper care, multiple emotions emerge while waiting for a solution, and preferences for direct specialist access vs general practitioner gatekeeping vary depending on individual and healthcare system. Substudy 3 developed visual representations of 5 evocative patient journeys, outlining stages, obstacles, interactions, and emotions.

**Conclusions:**

The journey of Europeans with MSK complaints is intricate and country specific, posing challenges for nontailored solutions. Personalised digital tools, improved healthcare provider education, and efficient communication between care levels are recommended to enhance the patient experience and improve outcomes for individuals with RMDs.

**WHAT IS ALREADY KNOWN ON THIS TOPIC?**

• People living with rheumatic and musculoskeletal diseases (RMDs) experience delays in diagnosis and care that affect their lives.

**WHAT THIS STUDY ADDS?**

• We have synthesised visually the experience of people with RMDs before arriving at a proper diagnosis based on data from multiple sources (including a systematic review, surveys, and focus groups) and then co-designing personas and journey maps.

**HOW THIS STUDY MIGHT AFFECT RESEARCH, PRACTICE OR POLICY?**

• The research can be used as framework for understanding the context and proposing and implementing digital solutions.The personas and their journeys resonate well with the lives of people with RMDs and, in addition to facts and numbers, can be used to develop policies and strategies.

Alt-text: Unlabelled box dummy alt text

## INTRODUCTION

More than a third of the global population may experience musculoskeletal (MSK) complaints [1]. Despite their significant prevalence and impact on quality of life, the general population has low awareness, and health professionals often have limited knowledge about rheumatic and MSK diseases (RMDs). These are usually not well diagnosed or treated, partly due to the symptom’s high prevalence, which normalises the experience, and the difficulty associating the symptoms with RMDs without proper training [1,2].

The persistent delay in diagnosing RMDs leads to postponed treatment and poor patient outcomes [[3], [4], [5], [6], [7], [8], [9]]. The average delay between symptom onset and treatment initiation in patients with rheumatoid arthritis (RA) is 12 months [7], which exceeds the 3-month window of opportunity for better outcomes [10,11]. In spondylarthritis (SpA) and fibromyalgia (FM), this delay is even longer, nearly 3 years in SpA and 6 years or more in FM [3,12]. The delay in FM diagnosis has been associated with an extensive use of medical resources and suffering [13].

A patient journey is the experience from admission to discharge in a healthcare setting or from help seeking to diagnosis, treatment, or even cure. It can be outlined for hospital stays, consultant appointments, or even standard check-ups with a general practitioner (GP) or nurse, and it follows a nonstandardised methodology to identify actors (as named in patient journey methodology, and we will call stakeholders from now on), events, and touchpoints [14]. A touchpoint is any moment of contact or interaction between a patient and the healthcare system, a technology, or an information source that is meaningful to their experience or decision making. Each touchpoint of the patient journey, from visiting a website to checking in for an appointment, has downstream effects on healthcare delivery, some of which may delay proper treatment.

The Stratification of Patients Using Advanced Integrative Modelling of Data Routinely Acquired for Diagnosing Rheumatic Complaints ((SPIDeRR) project is a European Union (EU)-funded project that aims, among other objectives, to improve the experience of people with RMDs through the healthcare system until they are offered proper treatment via digital tools [15]. Currently in development, these tools could potentially reduce the number of inefficient iterations between patients and the healthcare system, reduce the diagnostic delay, and accelerate the time to the best treatment option. Other studies of the SPIDeRR consortium are refining such tools and testing their validity and applicability [16].

The primary objective of this study within SPIDeRR was to comprehend the experience of people with symptoms of potential RMDs and their journey to a diagnosis and appropriate treatment, including who they meet in their journey, their interactions and interdependencies, events, points of contact, emotions, and bottlenecks. Secondary objectives were to investigate the challenges and opportunities within the patient journey for implementing digital tools to access the best treatment in rheumatology in a timely manner.

## METHODS

To better understand patients’ experiences and how the SPIDeRR tools can enhance them, we used the patient experience mapping framework derived from most patient journeys’ reasoning and steps [14,17,18]. These are based on the user experience (UX) methodology used by designers and rooted in creative design principles [17], such as the value-sensitive design [19], experience-based design by Barnett and Thomas [20], or design thinking visually [21]. The patient experience mapping framework helps identify touchpoints, events, obstacles, and opportunities for improvement, as well as stakeholders and their relationships, emotions, and interdependencies. It suggests the following steps: (i) identify key stages, (ii) define touchpoints, (iii) gather patient insights, (iv) create the map, (v) analyse and identify gaps, and (vi) develop solutions.

**Figure 1:**
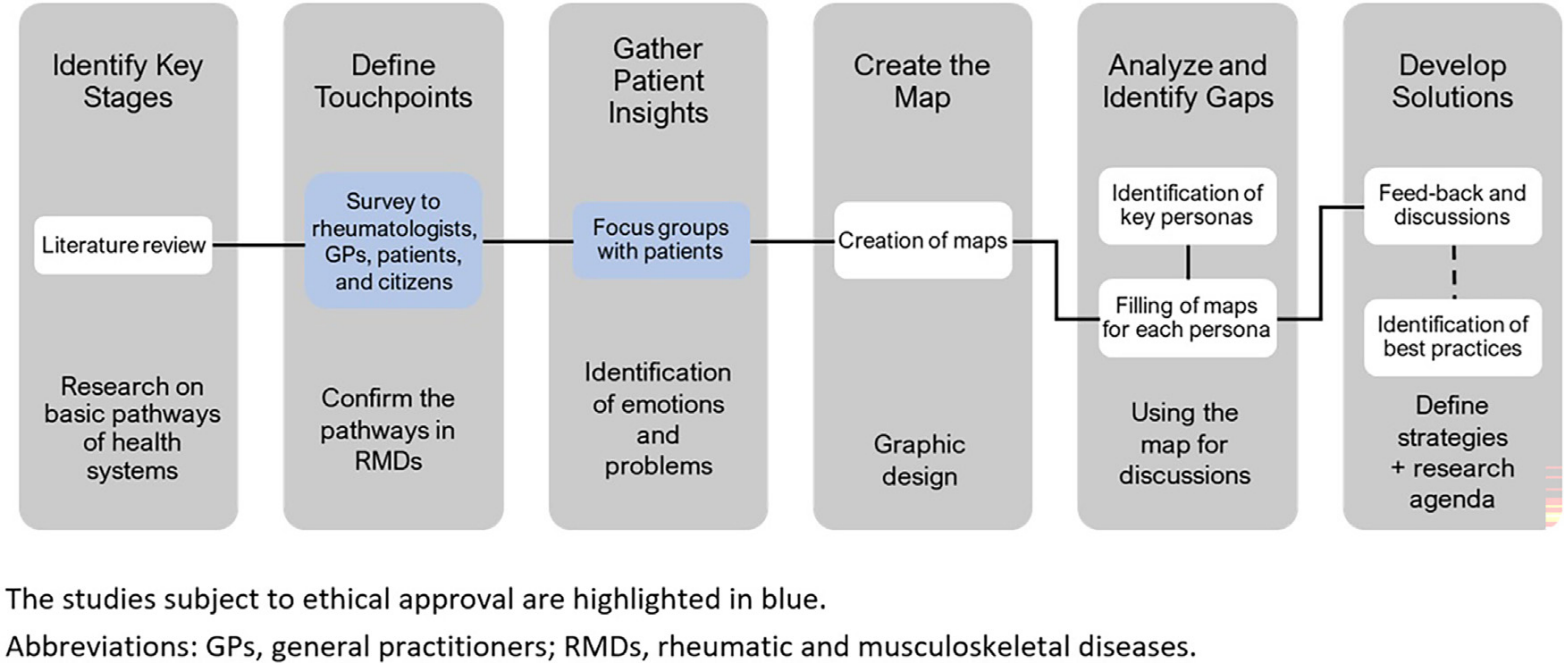
Substudies and activities of the project. The studies subject to ethical approval are highlighted in blue. GP, general practitioner; RMD, rheumatic and musculoskeletal disease.

This framework was translated into sequential and complementary studies and cocreation activities. The studies included a literature review, a survey of healthcare users and providers of the different health systems, and focus groups. The cocreation activities included the graphic design of the map, profiling of the personas, and discussions (Fig 1).

### Identifying key stages and defining touchpoints

This substudy aimed to identify critical aspects and touchpoints of the health systems of the SPIDeRR countries concerning the identification, diagnosis, and treatment of people with MSK complaints and RMDs. The SPIDeRR countries are Germany, Greece, Hungary, the Netherlands, Sweden, Spain, and the United Kingdom.

First, we gathered information via the following: (i) searches on repositories of health systems descriptions; (ii) a systematic review (SR) of published qualitative studies, leading to the development of patient journeys of people with RMDs; and (iii) an online survey that permitted to triangulate the information collected from the literature review into more precise steps and confirm them. The SR and the survey have been published elsewhere [22,23]; therefore, we present a summary in this study to help understand the findings and next steps.

As for the repositories searched, we identified institutional websites that inform about health system characteristics at the World Health Organisation (WHO), the Organisation for Economic Co-operation and Development (OECD), and the EU Commission levels. The results from publicly accessible reports are summarised.

The SR explored the journey of people with MSK complaints or RMDs from the perspectives of patients and healthcare professionals. Using qualitative synthesis methods [20], the review combined and interpreted findings from selected studies, peer-reviewed qualitative or mixed-methods research reporting patient and provider experiences from symptom onset to follow-up. Data were extracted with an adapted JBI form, capturing study characteristics and key aspects of the patient journey based on the patient experience mapping framework, such as journey phases, stakeholders, emotions, and context. Thematic framework analysis guided data synthesis [24]. One reviewer (TO) identified themes using a predefined structure [22], which was updated as new themes emerged. Themes were discussed, refined collaboratively, and categorised according to the prediagnosis phases described by Xiang et al [25]: (i) symptom onset until first visit to healthcare professionals; (ii) first visit to healthcare professionals until referral to rheumatology; (iii) rheumatology referral until first rheumatology visit; and (iv) first rheumatology visit until diagnosis. Findings were organised in tables to compare themes and subthemes, and results were mapped to the review’s objectives.

The survey, which has been published elsewhere [23], targeted rheumatologists, GPs, people with RMDs, and citizens without RMDs diagnosed, regardless of whether they had MSK complaints, from the countries involved in the SPIDeRR project. They were recruited via SPIDeRR’s network using the snowball technique. Each researcher proposed contacts from each target group in their respective countries. The contacts were then informed about the study, including objectives and timeline, and requested their consent before sending the survey. The questions were designed after the health systems’ documentation, and the SR were advanced. The main sections of the questionnaire included the following: (i) services and professionals available for people with MSK complaints in the public and private sectors; (ii) pathways to diagnosis; (iii) pathways to proper treatment, including specialised ones; and (iv) basic descriptors. We used descriptive summary statistics, for example, absolute and relative frequencies by Likert level and merging strata. The survey aimed at a sample size of 300 people, 10 per stakeholder per country. Given that the objective of the survey was not to gather opinions or carry out a statistical analysis, but to confirm the layout of the health systems, even 1 response per stakeholder per country would have been sufficient if this person had been well informed about the country’s health system.

### Gathering patient insights in focus groups

All the information gathered in the previous step was used as input for a qualitative study. The aim was to identify patients’ experiences and emotions through their interactions with the health system and other touchpoints in seeking a diagnosis and the best treatment for MSK complaints.

Four focus groups, each 1.5 hours via Zoom, were conducted in English by experienced qualitative researchers with a background in rheumatology, both from Spain (TO and LC). The graphic designer outlining the patient journeys (MP), who had experience with the patient journey methodology and lived in Germany, attended as an observer.

The focus groups targeted persons from the public, with and without RMDs, from the countries involved in the SPIDeRR project. A profile grid was established to ensure at least 1 representative in each stratum: age (<30, 30-50, and >50 years), gender (male and female), and disease (no RMD, inflammatory RMD, and noninflammatory RMD). The sample size of the participants was justified by the number of people needed to complete the grid, resulting in a total of 18. Explanatory flyers in the local language were used to recruit participants via the SPIDeRR consortia networks and patients’ groups (eg, ReumaNederland, Deutsche Rheuma Liga, and Newcastle Hospital patient advisory groups). The rheumatologists and GPs in the team were also encouraged to recruit participants, directly or on social media. The flyers informed potential participants about the focus groups’ objectives and timeline and requested their consent to participate.

A guide helped orient the discussions (Appendix B.1). The participants were asked what they would do if they had an MSK problem in their country (non-RMD participants) or what they would have done better (RMD participants). Knowledge of the system, help-seeking behaviours, and emotions across all journey phases was purposefully inquired. In addition, we framed the data collected on the patient experience mapping framework as follows: (i) phases of the patient journey (awareness, help seeking, primary care, diagnosis, treatment, and follow-up); (ii) touchpoints/stakeholders (ie, the stakeholders who appeared in the journey, such as the patients themselves, GP, rheumatologist, or others); (iii) emotions; and (iv) context, more concretely, disease or health problem, and country.

The sessions were recorded for collection and subsequent analysis. One team member transcribed the audio files, noted relevant observations, and deleted them. The transcriptions bore no identifiable information and were used for the qualitative analysis. Data were coded and analysed using a mixed inductive (based on the themes and subthemes that emerge) and deductive (based on the points needed for the journey map: stakeholders, relationships and interdependencies, events, points of contact, emotions, and obstacles/opportunities) technique using the ATLAS.ti software (ATLAS.ti Scientific Software Development GmbH, 2018) for qualitative analysis.

### Creation of the journey maps and personas

With the information collected and analysed from the previous steps, the patient journey maps were cocreated with the graphic designer’s (MP) guidance. The cocreation platform Miro was used for the iterations in the outlining group [26]. All SPIDeRR members were invited to a 2-hour creative session in which they could create personas based on the critical attributes identified in the previous steps. Personas are character profiles that represent a group of actual healthcare users, but they are not real people. They also describe behavioural patterns, goals, skills, and attitudes [27]. In this case, they represented people with MSK symptoms and various complex journeys, extracted from the previous research and the experience of our patient research partners and representatives.

The steps to create personas were those defined by the Interaction Design Foundation (IxDF) [28]. First, we used the information from the focus groups about general interests, feelings, and actions on each key stage. A long list of attributes, from behaviours to personality traits, was displayed on a Miro board. The participants were instructed on how to sketch a persona and that they could be based on a real person. Participants began filling in basic information such as age, gender, and socioeconomic status and then delving more deeply into the personality and story using attributes for the personas. These attributes, or ideas guiding the stories, could be used to create a full view of the persona. They were presented in categories, such as realistic, investigative, artistic, social, enterprising, or conventional (Appendix B.10). Participants were instructed not to duplicate certain attributes by marking them as used to avoid overrepresenting them.

In addition to the attributes, the personas were completed with information on their living and work situations (eg, Who do they live with? Do they have to take care of someone? Do they have someone who takes care of them? Do they live in a rural or urban environment? How easy is it to get a doctor's appointment? What’s their availability for appointments? Can they take a few hours away from their jobs, or is it not easy?), their relationship with the healthcare system (eg, Have they been around it before? How was their experience so far? Does anyone in their family have illnesses? Has that affected their view of the system? Do they have acquaintances and/or relatives working in healthcare? Do they have any other non-MSK disease?), and some relevant behaviour issues (Do they like a self-management approach or prefer straightforward instructions? How does their technology use look? Do they go on the Internet regularly? Are they good with computers? Do they seek help or go to the doctor themselves or is someone telling them to do it?). Then, we sketched as many profiles as the workshop participants considered necessary by selecting attributes obtained from the previous step. Finally, we decided to create 5 personas. The profiles were captured in a short description, balancing data and knowledge about real applications with fictitious information intended to evoke empathy. The description included information about social background, psychological characteristics, and emotional relationship with the focus area. After completing the persona profiles, they were presented to the researchers and patients from the team to receive their input and agreement through the Miro platform. We also circulated the personas among participants in the focus groups for validation, instructing them to identify inconsistencies and crosscheck with their countries’ realities and experiences of people they know. In the end, we used the information gathered about the experiences to understand the persons and to predict what actions they will perform.

## RESULTS

### Identifying key stages and defining touchpoints

#### EU and OECD reports

We identified key reports with health systems descriptions, Health at a Glance: Europe 2022 [29], the State of Health in the EU Synthesis Report 2023 [30], and the Health Systems Information Survey [31], which in turn are based on Eurostat surveys and others. In summary, Europe is a privileged region with sound health systems that ensure a high life expectancy, from 72 to 84 years [29]. Table 1 synthesises the country health profiles developed by experts from the OECD and the European Observatory [32]. The Table also includes data from the 2023 round of the OECD Health Committee survey on Health System Characteristics, which has a publicly accessible dataset [31].

**Table 1:** Synthesis of the country health profiles developed by experts from the OECD and the European Observatory and the Health Systems Characteristics Survey

Country	Life expectancy	Health spending^a^	Funding structure	Resources/workforce	System structure/focus	Key challenges
Germany	80.7	5159	Public funding OOP 12% Main OOP areas: long-term care and pharmaceuticals	High numbers of healthcare professionals and hospital beds per capita Shortage of hospital nurses	Extensive hospital sector Hospital care is the largest expenditure	Shortage of hospital nurses High hospital usage prompting reforms
Greece	80.7	1874	High OOP payments	Healthcare staff shortages	Introducing gatekeeping and care integration systems	High OOP is causing unmet needs and financial hardship High rates of missed care, staff shortages, and quotas on public services
Hungary	76.2	1382	Compulsory insurance covers 95% OOP 25% (nearly half spent on pharmaceuticals)	Hospital bed densities above the EU average Workforce retention is challenging (private sector and emigration)	Hospital-centric system	Rapidly ageing population with chronic conditions. High OOP Workforce retention challenges
Netherlands	81.7	7179	Private sources contribute 15% of total spending	Fewer doctors and more nurses per capita than the EU average GP shortages	Long-term care is 28% of spending, exceeding EU averages	Worsening GP shortages expected Hospital nurse shortages
Sweden	83.1	4200	Public funding covers 86% User fees capped annually, with exemptions for specific groups	Generally good access Remote regions face access issues	Universal coverage includes free or low-cost services with copayments	Access issues in remote regions Increased elective surgery waiting times
Spain	83.2	3234	Public funding 98% Increasing voluntary insurance OOP exceeds the EU average Financial solid protections exist	Shortages of healthcare workers, especially in rural areas	The primary care system excels in managing chronic conditions	Multimorbidity Workforce shortages, especially in rural areas OOP exceeds the EU average
United Kingdom	81.4	5738	OOP 15% Main OOP areas: dental services, prescriptions, and personal care	High ratio of healthcare professionals and hospital beds per capita	Focus on hospital and primary care services	Staffing shortages

EU, European Union; GP, general practitioner; OECD, Organisation for Economic Co-operation and Development; OOP, out-of-pocket.

a In € (2021) per capita.

Regarding healthcare coverage, Spain and the United Kingdom have universal national public health systems covering the total population, Greece and Hungary have a single health insurance fund, and Germany and the Netherlands have multiple funds or companies. Sweden is organised into local health systems. As to the comprehensiveness of basic healthcare coverage, inpatient and outpatient care and laboratory or imaging tests are free in Spain, Hungary, and the United Kingdom. Some or all concepts are deductible in other countries or a small fee applies. The situation is more complex regarding medicines. Even in countries where health is universal, there is some copayment.

All countries provide free access to primary care, with small user fees or copayments in Greece and Sweden. The GP is a gatekeeper in Spain, Hungary, the Netherlands, and the United Kingdom, where GP referral is compulsory to access most specialist care (except in case of emergency). In all others, there is more flexibility, especially in Greece.

Finally, regarding the digitalisation of health systems, all primary care delivery systems use health data systems or electronic health records, with the United Kingdom, Netherlands, and Spain having the most complete deployment of health information systems (Appendices B.2-B.8 for the specific results of the Health System Characteristics survey). With exceptions, care is fragmented between primary care and rheumatology.

The proportion of individuals aged 16 to 74 years making an appointment with a clinician via a website has increased from 2012 to nearly 40% in 2022; however, the level of education may be a determinant [30]. In 2021, the share of people aged 16 to 74 years who had at least basic overall digital skills across the EU varied between 79% and 28%, with an average of 53.92%. The SPIDeRR countries are spread within that range, with the Netherlands having the highest percentage of its population with basic digital skills (79%,), followed by Sweden (66.60%), Spain (64.16%), Greece (52.48%), Hungary (49.09%), and Germany (48.92%) [33]. In the case of UK citizens, a 2018 study reported that 79% of the population had the full basic digital skills [34].

#### SR of journeys of patients with rheumatic diseases

The SR includes 36 studies of acceptable quality. Among the included studies, 6 studies focused on symptoms or general RMDs (including arthralgia, MSK pain, and autoimmune diseases) and 29 on specific conditions—10 on RA, 1 on early RA, 5 on osteoarthritis (OA), 6 on SpA, 4 on FM, 1 on gout, and 3 on rare diseases (inclusion body myositis, lupus, and polymyalgia rheumatica) [22].

After the thematic analysis, the first result is the redefinition of the phases of Xiang et al [25]. Instead of the following—(i) symptom onset until first visit to healthcare professionals; (ii) first visit to healthcare professionals until referral to rheumatology; (iii) rheumatology referral until first rheumatology visit; and (iv) first rheumatology visit until diagnosis, we realised that usually the journeys include a split of the phases into the following: (i) awareness, (ii) help seeking, (iii) first encounter, (iv) clinical suspicion in primary care, (v) diagnostic tests in primary care, (vi) management (outside of rheumatology), (vii) rheumatology referral, and (viii) management in rheumatology.

The touchpoints or critical stages of interaction within the healthcare system, were as follows: (i) awareness—not a touchpoint strictly speaking, but critical; (ii) help seeking; (iii) first encounter with a healthcare professional (usually a GP, but can be other healthcare professionals); (iv) clinical suspicion in primary care; (v) diagnostic tests in primary care; (vi) management outside rheumatology (eg, by a GP); (vii) rheumatology referral; and (viii) management in rheumatology.

The stakeholders who play a role in the patient journey are the patients themselves and their families, their social and work relationships, the health system, the GP, physiotherapist, orthopaedic surgeon, and other healthcare professionals dealing with MSK symptoms, rheumatologists, and other specialists. The most significant emotions mentioned by the patient during their journey in the included articles were frustration, acceptance, coping, and trust (in the doctor-patient relationship, improving management and outcomes). Common themes were the myriads of healthcare professionals visited (especially people with FM or back pain), the delays in referral, diagnosis, and treatment, suboptimal management, and the lack of a truly patient-centred system.

Moreover, several contextual factors influencing the patient journey are highlighted throughout the studies, such as health literacy and education; the patient’s families, social, and work relationships; the system favouring pathways or blocking access; the knowledge and recognition of the disease by the healthcare professionals; appropriateness and flexibility of protocols in primary and specialised care; limited access to rheumatology; and the coordination between healthcare levels and specialists.

#### Survey of health systems of SPIDeRR countries

The sample comprised 141 respondents from the general public, 67 rheumatologists, and 39 GPs. It was acceptably balanced across the countries and stakeholder groups. A summary of the main findings are described further [23].

The most likely initial touchpoints for people with MSK symptoms were searching the internet directly and going to a GP consultation. This pattern was similar across participant groups and countries. Internet searches significantly influence patient behaviour and expectations during GP visits.

In almost all countries, GPs act as gatekeepers for rheumatology referrals, except in Greece. In countries such as Hungary, there was inconsistency between GPs and rheumatologists on whether this role is mandatory. For all countries, both GPs and rheumatologists generally rated GPs’ knowledge of RMDs as moderate to low, with the highest knowledge in Sweden and the lowest in Hungary.

Access to rheumatologic diagnostics, diagnosis time, and treatment varied widely across countries. In Hungary and Spain, access to sacroiliac magnetic resonance imaging (MRI) was limited. Glucocorticoids were widely available, while disease-modifying antirheumatic drugs were more accessible in Germany and Greece. Access to physiotherapists and psychologists was notably lower in Hungary, Greece, and Spain. Specialist nurses and occupational therapists were generally limited, except in the United Kingdom. Public service access was typically higher than private.

Access to rheumatology services varied by country. Healthcare users found it hardest in Spain, the United Kingdom, and Germany, whereas GPs considered it most difficult in Germany and easiest in the United Kingdom. From the perspective of rheumatologists, there were challenges in Germany, and almost none in Greece. Healthcare providers reported that the United Kingdom had the highest access to specialised units, whereas Greece and Hungary had the lowest. Early arthritis clinics were moderately to highly available, with the United Kingdom being the most accessible. Triage in rheumatology was standard in the Netherlands, the United Kingdom, and Sweden, frequent in Germany, but less so in Spain, Hungary, and Greece.

Diagnostic delays for rheumatology varied significantly between stakeholders. Healthcare users reported the longest delays in Spain and the shortest in Sweden. GPs saw Germany as having the longest delays, and Greece and Sweden, the shortest. Rheumatologists viewed the Netherlands as having the shortest delays, while Germany and the United Kingdom as having the longest.

All respondent groups viewed healthcare systems as best organised in Sweden, the Netherlands, and the United Kingdom, and poorest in Greece and Hungary. As to the healthcare providers’ responses, direct communication between care levels was limited, often relying on letters or patients. Rheumatologists noted direct communication taking place mainly in Sweden and, to a certain degree, in Spain and the United Kingdom. One-way communication, such as specialist notes to GPs, was possible in all countries.

#### Gathering patient insights in focus groups

Twenty-eight people participated in the focus groups, of whom 17 (61%) had an RMD (12 inflammatory and 5 noninflammatory), and 18 (64%) were women. Participants’ ages were distributed as follows: 14% (n=4) were <30 years, 54% (n=15) were between 30 and 50 years, and 32% (n=9) were >50 years. They came from all participating countries: Germany (4), Greece (4), Hungary (3), Netherlands (4), Spain (3), Sweden (2), and the United Kingdom (6).

**Figure 2:**
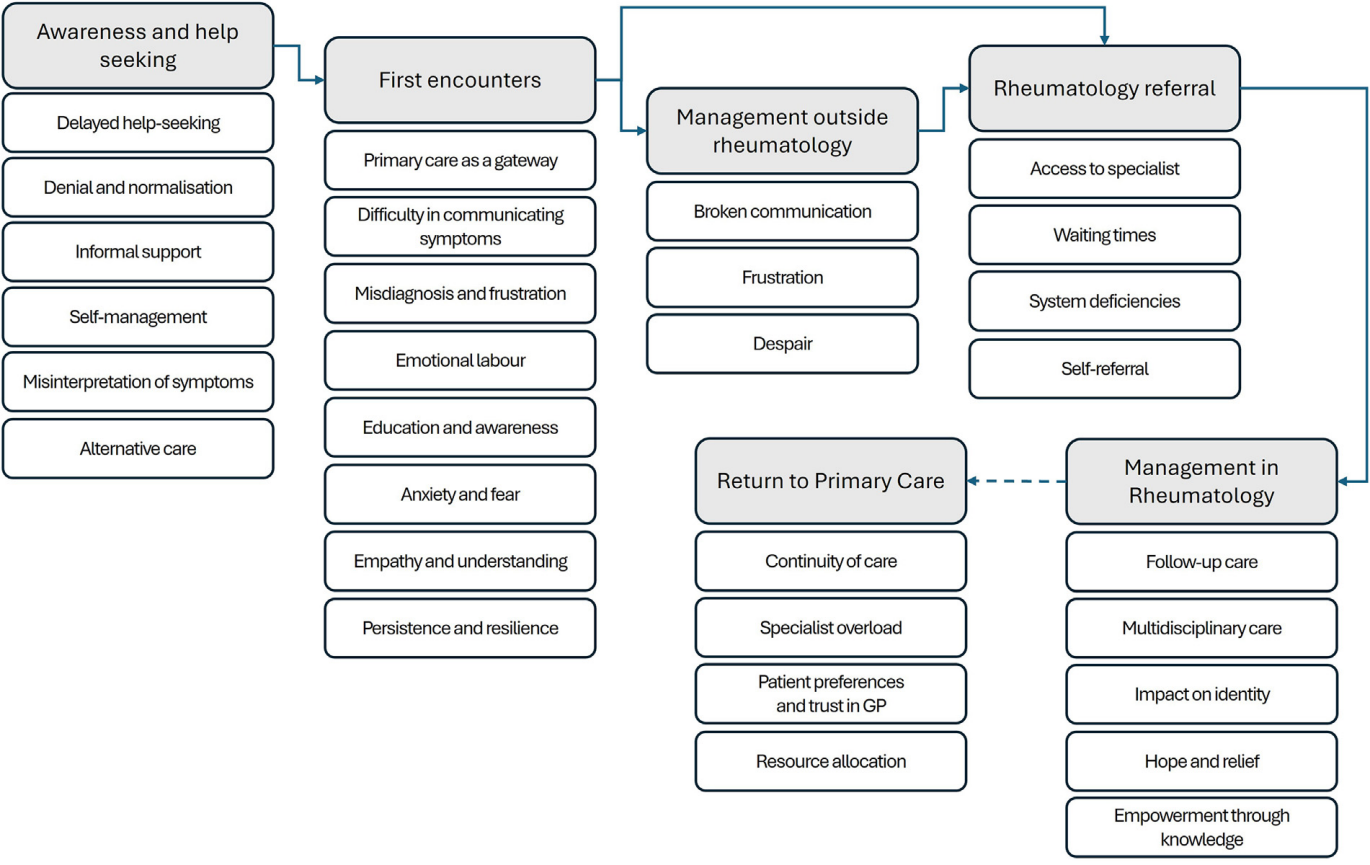
Phases of the journey of people with rheumatic and musculoskeletal diseases and the major themes aroused at the focus groups.

The themes that emerged in the different phases of the journey are detailed further and in Figure 2. A table B.9 in Appendix B shows examples of supporting participants’ quotes. Note that after the discussions, another phase emerged: going back to the GP.

#### Awareness and help-seeking phase

Many participants initially delayed seeking medical help, often normalising their symptoms or waiting until the pain became severe before consulting a healthcare professional. Patients may not recognise the significance of their symptoms early on, leading to delays in seeking medical attention. Some participants, especially those without prior experience with MSK conditions, said they would initially wait a few days to see if the pain subsided before contacting a doctor. This behaviour may be influenced by a belief that the symptoms are not severe enough or by a reluctance to visit the doctor unless necessary (delayed help seeking).

There was a tendency among individuals to deny the severity of their symptoms or to normalise them as part of ageing or lifestyle, which could lead to a delay in seeking medical help. This denial could also be a coping mechanism to avoid facing the potential implications of their symptoms (denial and normalisation).

Others would research online and consult with family members or friends who work in healthcare for advice on the next steps. Some individuals relied on informal support networks, such as family members with medical expertise, for initial advice and symptom management. This approach was taken before or in conjunction with seeking formal medical care (informal support).

Without proper medical knowledge, patients may misinterpret their symptoms, potentially leading to incorrect self-diagnosis or dismissal of serious health issues (misinterpretation of symptoms).

Participants initially tried to manage their symptoms independently, ignoring the problem or attributing it to other causes, such as overexertion or lifestyle factors. This self-management included avoiding activities that exacerbated the pain or trying to function normally despite the discomfort. Those with a background in healthcare or related fields felt they could self-diagnose and start appropriate exercises, only seeking medical attention if the pain persisted or worsened (self-management).

Some participants were prescribed or sought out complementary therapies, such as acupuncture, as part of their initial symptom management. These therapies provided temporary relief but did not lead to a definitive diagnosis or cure (alternative care).

### First encounter, clinical suspicion, and tests in primary care

The GP was often the first point of contact for participants seeking help for their symptoms. GPs would then refer them to specialists or recommend further investigations, such as X-rays or blood tests (primary care as a gateway). The GP’s role in health system navigation is crucial. They serve as the first point of contact for many health issues and are responsible for referring patients to specialists when necessary. The effectiveness of this gatekeeper role varies, with some participants expressing trust in their GPs’ judgement, while others feel they need more control over accessing specialists (role of the GP).

Some participants discussed the difficulty of accessing specialists immediately when symptoms arise, which can lead to prolonged periods of uncertainty and untreated pain (lack of immediate access).

Individuals had to persist in advocating for themselves as they faced scepticism or dismissal from healthcare providers. This persistence was crucial in eventually leading to a correct diagnosis and appropriate treatment. Despite the challenges, participants also displayed resilience and persistence in their quest for a diagnosis and effective treatment. This determination could be emotionally draining but empowering as they advocate for themselves (persistence, advocacy, and resilience).

The journey to a correct diagnosis was often fraught with misdiagnoses and frustration. Participants experienced being told that their symptoms were due to laziness or other nonrheumatic causes, leading to delays in appropriate care (misdiagnosis and frustration).

The constant need to explain and justify their symptoms to healthcare professionals could be emotionally taxing. Participants had to present their case convincingly to ensure they received the needed care (emotional labour).

The uncertainty surrounding the cause and progression of symptoms could lead to anxiety and fear. Participants worried about the impact on their functionality and the potential for a chronic condition (anxiety and fear).

The discussion underscored the need for better education among healthcare professionals regarding the early signs of rheumatic diseases. Participants felt doctors should be more aware of the potential for autoimmune diseases and better equipped to recognise and investigate these conditions (education and awareness).

A lack of medical education can make it challenging for patients to effectively communicate their symptoms to healthcare providers, hindering accurate diagnosis (difficulty in communicating symptoms).

Participants emphasised the importance of empathy and understanding from healthcare providers. They desired a more patient-centred approach, considering the individual’s experience and perspective (empathy and understanding).

### Management outside rheumatology

Participants often expressed frustration due to the lack of a clear diagnosis, healthcare professionals’ dismissal of their symptoms, and slow progress in finding relief. The frustration was compounded by the feeling of not being taken seriously (frustration).

Participants had differing perspectives on the ideal patient pathway. Some preferred directly accessing specialists such as rheumatologists without needing a referral from their GP, as it occurs in Greece. Others felt the GP should act as a gatekeeper, providing an initial assessment and referring to specialists as needed. However, frustration was expressed about the long wait times to see specialists through the public healthcare system. The experience of chronic pain and the challenges of managing symptoms could lead to feelings of despair. Participants might feel misunderstood by medical professionals and even friends and family who cannot fully grasp the extent of their discomfort (despair).

Building trust and communication with the primary care provider was important in determining the preferred patient pathway. There was a call for improved communication between medical specialities, healthcare providers, and patients. Better coordination and shared decision making were essential for more efficient and effective symptom management and treatment (improved communication).

### Rheumatology referral

One of the primary challenges discussed is difficulty accessing specialists, particularly rheumatologists. As mentioned before, GPs play a decisive role as gatekeepers, meaning patients must first consult their GPs before being referred to a specialist. This can lead to delays in diagnosis and treatment, as mentioned by several participants (access to a specialist). Long waiting times for appointments with specialists and diagnostic tests are a common frustration. This is particularly problematic for those with chronic conditions that require accurate diagnosis, specific therapy, and disease management and monitoring (waiting times). Participants mentioned that these long waiting periods to see a specialist could be due to the healthcare system’s structure or the limited availability of specialists (system deficiencies).

In some countries, such as Greece, patients can directly consult specialists without first seeing a GP. This can streamline the process for those who suspect they need specialised care. However, there is a concern that this might lead to overcrowding of specialists with cases that GPs could manage (self-referral).

### Management in rheumatology

After seeing a specialist, there is often a debate about who should manage the follow-up care. Some participants feel comfortable with GPs managing their condition once a specialist has provided a diagnosis and treatment plan. In contrast, others prefer continued specialist care due to the complexity of their condition (follow-up care).

The onset of symptoms could affect participants’ sense of identity, particularly if they were used to being active and independent. For example, participants described the need to adjust to physical limitations and the uncertainty of the future as identity altering (impact on Identity). Some participants were not initially aware that their condition being inflammatory could mean chronic. The chronic nature of these conditions was more worrying than the specific diagnosis. They thought an inflammation would only be temporary and were surprised when the symptoms persisted. Moreover, the term ‘degenerative disease’ was concerning to some participants, who associated it with more severe conditions. Better communication from healthcare providers about what the diagnosis means is needed. While the eventual diagnosis can be daunting, it can also bring relief and hope. Knowing the cause of the symptoms allows participants to seek appropriate treatment and support (hope and relief).

The idea of multidisciplinary teams that can holistically address a patient’s health is seen as a potential improvement. This approach could empower patients by providing a more comprehensive understanding of their condition and treatment options (multidisciplinary care).

As participants became more informed about their conditions, they often felt empowered to make better health decisions and communicate more effectively with healthcare providers (empowerment through knowledge). There is a strong emphasis on patient education and self-management. Participants value being informed and having the tools to manage their health independently. This includes using applications and online resources to monitor conditions and make informed decisions. Good health education, for example, provided by patient associations, can increase patients’ ability to make informed decisions about their healthcare, potentially leading to a more active role in their treatment (patient education and empowerment).

### Going back to the GP

Participants understood that rheumatologists may refer patients back to their GPs for disease management in order to reduce costs and optimise the healthcare system (specialists’ overload). The back referral to the GP was felt as a healthcare system’s approach to allocating resources, where specialists focus on complex cases and GPs handle routine follow-up and management (resource allocation).

Some participants expressed disappointment at the prospect of being discharged from rheumatology care. They said they would trust the specialist’s judgement more than a GP when follow-up is needed. It might be acceptable for the GP to manage follow-up care after a specialist establishes a diagnosis and initial treatment plan (continuity of care).

Depending on the complexity and severity of their health issue, some patients may prefer to continue with a specialist, whereas others may be comfortable with their GP managing their condition. The level of trust and familiarity with one’s GP can influence the acceptance of being referred back to the GP for ongoing management (patient preferences and trust in GP).

### Contextual factors

Some contextual factors were cross-sectional, influencing all phases (Table B.10 in Appendix for supporting quotes). Healthcare systems differ significantly across countries. For example, participants mentioned that, in Hungary, nurses play a vital role in patient care, while, in the Netherlands, GPs are overworked and have limited time for individual patients. These differences influence how patients navigate their health systems (crosscountry variations). Some healthcare systems may prioritise certain conditions or patients over others, which can delay those seeking care for rheumatic diseases from receiving the care they need, because the system does not consider their condition urgent (health system prioritisation).

The cost of healthcare and the sustainability of the system are also considerations. There is an understanding that the referral process and follow-up care are partly designed to manage costs, but this can sometimes compromise the quality of care received by individual patients (cost and sustainability). The discussion touched on financial considerations, where the cost of specialist consultations or tests can be a barrier to accessing care, particularly in systems where patients must (partially) self-pay for such services (financial constraints).

Effective communication between healthcare providers and patients is essential for successful health system navigation. Patients want clear explanations of their conditions, treatment options, and the reasons behind the referral decision (communication and transparency). Delays can also stem from poor communication between healthcare providers, leading to inefficiencies in the referral process and potentially unnecessarily prolonged waiting times (communication and coordination).

Access to specialists can be influenced by where a patient lives. Rural or remote areas often have fewer specialists available, leading to longer travel times or reliance on telehealth services, sometimes seen as a facilitator and, in other instances, as a barrier to proper care (geographical barriers).

Additionally, patient characteristics and personality may influence the journey. Rheumatic diseases can present with varied and sometimes nonspecific symptoms, which can complicate the diagnostic process and lead to delays in identifying the need for specialist care (complexity of symptoms). The patients’ ability to advocate for themselves and their understanding of the healthcare system can impact the timeliness of accessing specialists. Some patients may not be aware of the need for specialist care or may not know how to navigate the system effectively (patient advocacy and knowledge).

#### Creation of maps and personas

**Figure 3:**
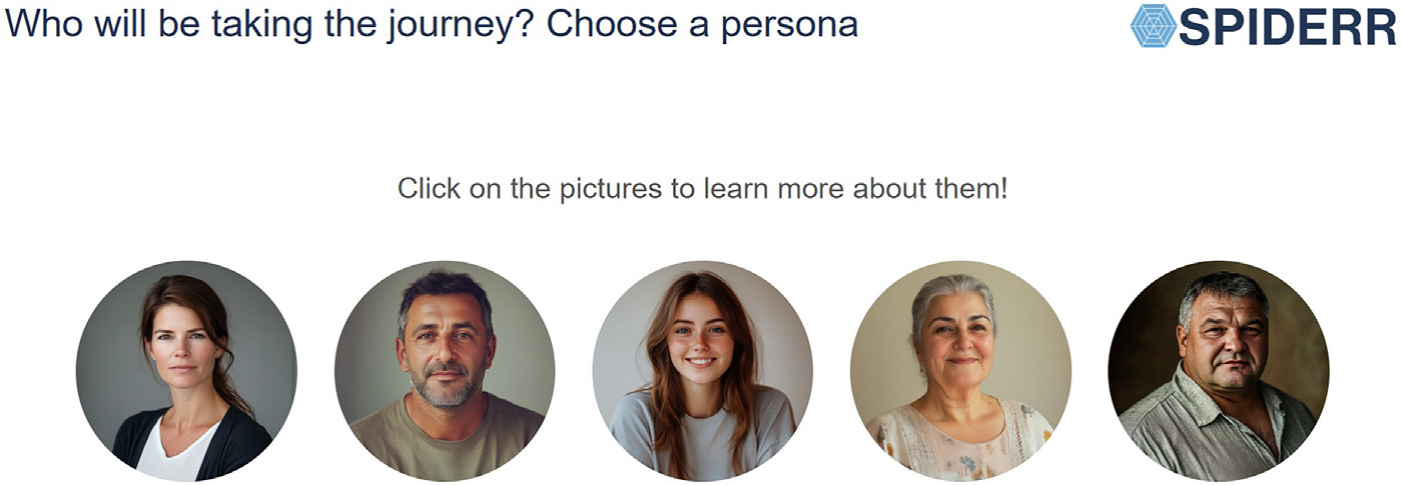
Screenshot of the second page of SPIDeRR’s interactive online journey maps, where one must choose a persona to display a journey map.

**Figure 4:**
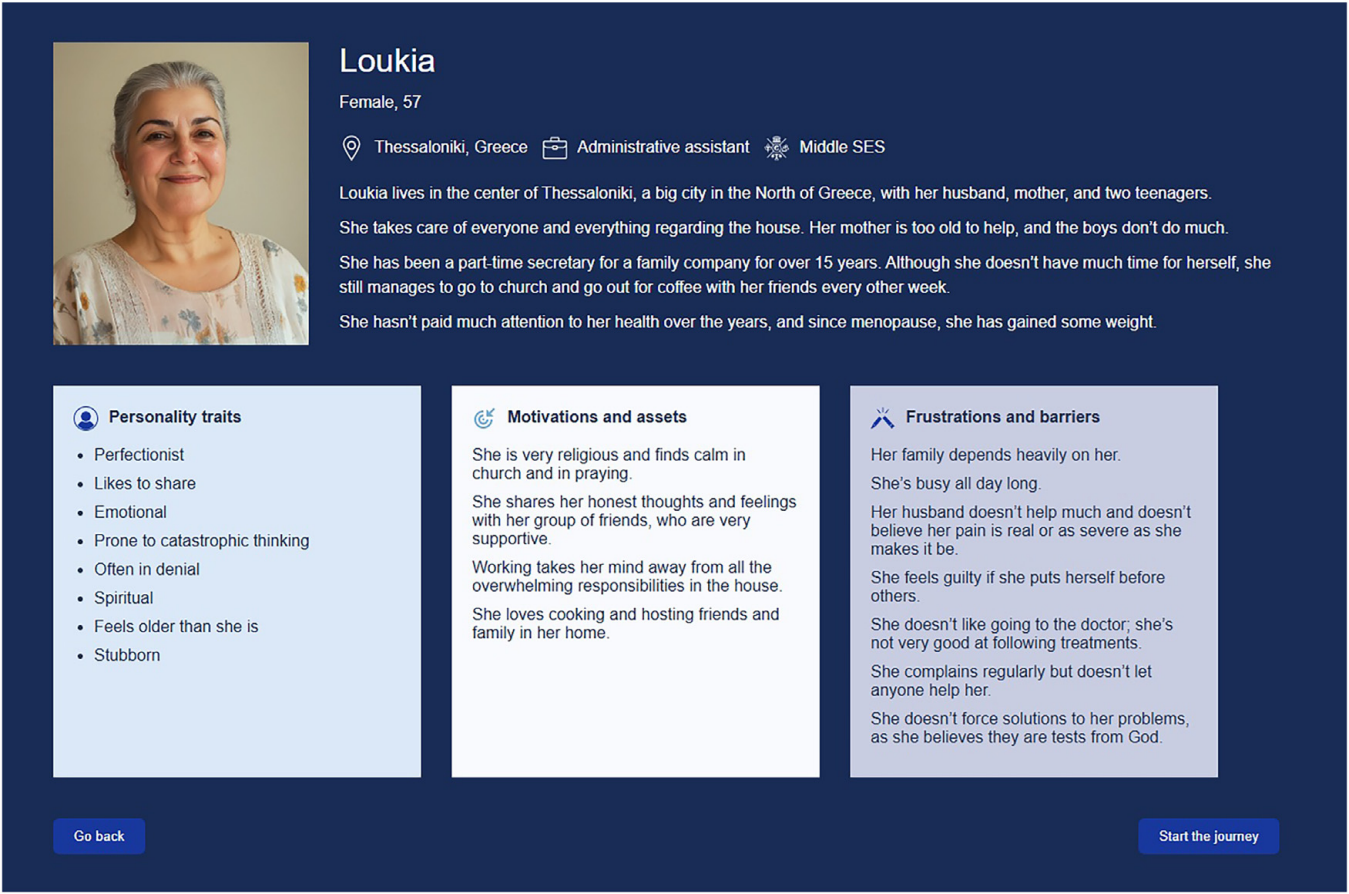
Screenshot of the description page of a persona.

**Figure 5:**
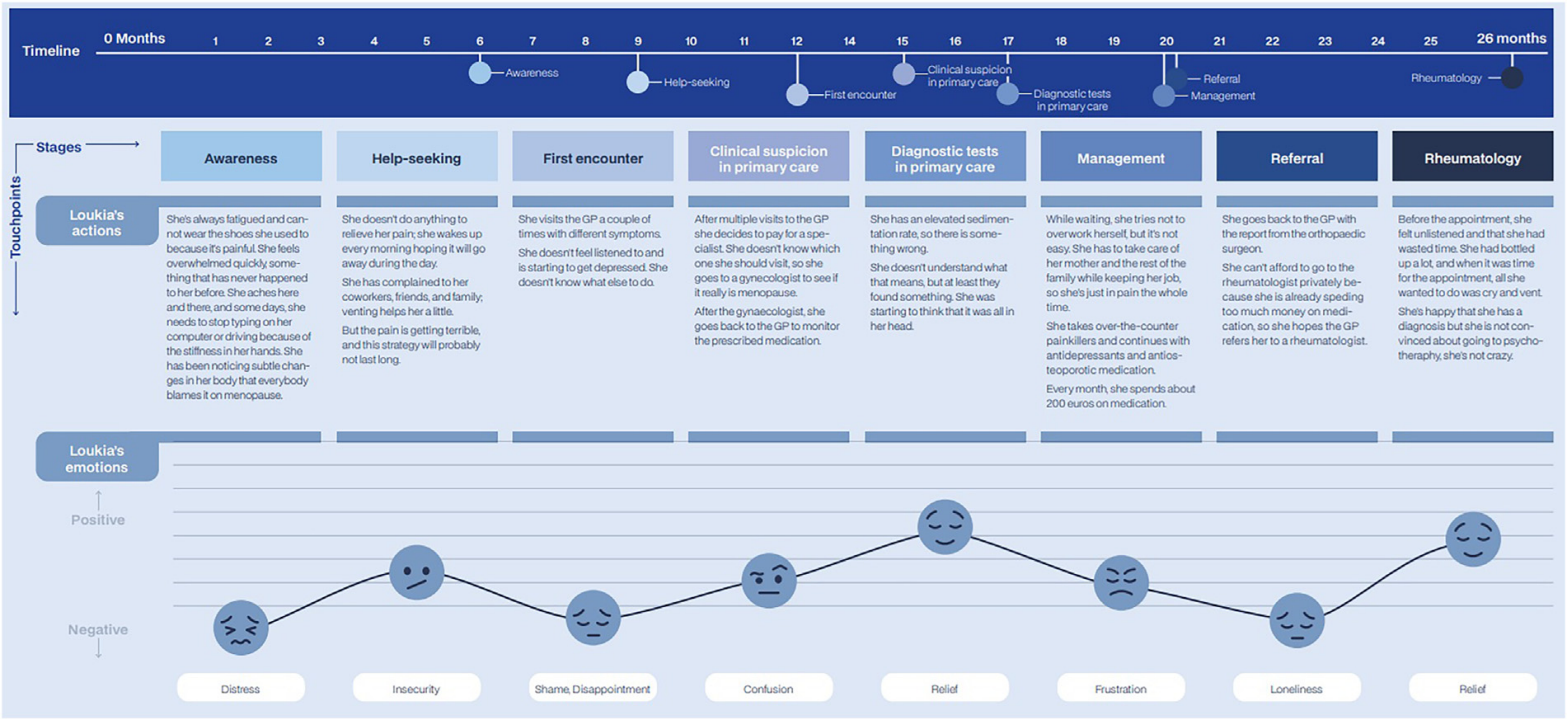
Screenshot of part of a PDF that can be downloaded to see the journey of Loukia.

The creation of personas started with a workshop attended by 19 SPIDeRR researchers and patient partners. The personas workshop resulted in the sketched preliminary profiles of 12 personas, which were subsequently consolidated into 5. The results were then transferred into patient journey maps that were reviewed by participants of the focus groups from the respective personas’ countries. The journey maps underwent several rounds of crosschecks for contradictions or counterintuitive steps by the patient advisory group and 1 researcher from each country. Five final journey maps were uploaded to an interactive webpage and are freely accessible (Figure 3, Figure 4, Figure 5; https://spiderr-project.eu/en/Interactive_Patient_Journey/)

## DISCUSSION

The patient journey approach can be used to assist the design, implementation, and monitoring of health services or tools by adjusting and refining the patient experience map to reflect improvements. They have been applied to better understand patients’ experiences and how the SPIDeRR tools can improve them, visually representing the patient’s journey from initial contact with the healthcare system to posttreatment follow-up.

Mapping patient journeys is challenging due to a lack of clear frameworks. The patient mapping experience framework that we used was not well-defined, so we had to adapt our learning to apply it systematically. Basically, its pillars are collecting data about the journey from different sources, visualising the UX, and structuring the data gathering and visualisations into phases, stakeholders, emotions, and context. New frameworks aim to integrate patient experience into real-world study design [19], with efforts underway to develop quantitative patient journeys within the SPIDeRR consortium. These quantitative journeys, or trajectories, relate more to clusters of symptoms or variables and the times spent in the phases of the journey based on data from electronic health records [35,36].

Personas—fictional characters based on study findings—help identify key user needs, promote patient-centred thinking, and improve resource usability by fostering empathy [37]. They offer teams a clear mental model of healthcare users, making it easier to predict behaviour and avoid projecting personal biases. For personas to be effective, they must feel like real people with relatable goals. In the technology-focused SPIDeRR project, personas have helped refocus discussions on patient needs. These learnings are helping guide the recruitment for studies to test the tools and develop tailored engagement strategies for those who would benefit most from our tools.

The documentation for the journeys showed us that healthcare access for patients with RMDs is challenging in Europe, despite all advancements. While the internet and primary care are key initial touchpoints, there are discrepancies in how healthcare users and providers perceive access and pathways. Challenges include GPs’ perceived limited knowledge of RMDs, limited access to specific diagnostic tests such as sacroiliac MRI in some countries, and difficulty obtaining rheumatology referrals. The shortage of rheumatology services and long waiting times are key factors limiting referrals. The variability in responses between GPs and rheumatologists regarding access to tests and services also reflects potential miscommunication between healthcare levels.

At the end of the focus groups, participants suggested ways to improve the patient experience, including empowering patients through education, self-management tools, and peer support; improving coordination between GPs and specialists; and expanding access to rheumatologists. They also recommended exploring direct access to specialists, increasing specialist numbers, using technology for monitoring and communication, offering tailored exercise and support programmes, and establishing multidisciplinary teams for holistic care. Addressing the emotional impact of chronic MSK diagnoses through counselling and support groups was also emphasised (Appendix B.9 has a description of the strategies proposed in the focus groups for improving healthcare).

The study may have some limitations. While we acknowledge that no recruitment strategy fully eliminates selection bias, the diversity of our channels – spanning clinical, community, and advocacy settings – was intended to produce a sample that reflects the broader population of sufferers, including those who are less activated or proactive. This is reflected in the persona set itself, which includes Emre or Laszlo, who were not proactively seeking help. Moreover, country selection was determined by consortium membership rather than theoretical sampling, which limits the generalisability of findings beyond high-income European healthcare contexts. Although the participating countries encompass variation in health system organisation, certain European regions remain unrepresented, and the patient journeys described may not reflect experiences in lower-income or non-European settings. Future research should use purposive crossnational sampling to examine how health system typology shapes the RMD patient journey. Finally, while 5 personas cannot exhaustively represent all patient scenarios in highly prevalent diseases, their attributes were systematically derived from qualitative insights, refined through iterative merging of multiple proposals, and validated across both patient and health system perspectives. Ongoing work within the SPIDeRR project will further ground these personas in real-world trajectory data (not available at the time of persona development), enabling empirical refinement of the patient journeys.

The SPIDeRR project aims to improve the patient’s journey by reducing diagnostic and proper treatment delays with artificial intelligence (AI)-driven tools. However, we cannot directly tackle the obstacles identified in the aforementioned journey. We cannot improve the shortage of professionals, waiting times, or other system bottlenecks. However, we hope to improve navigating the healthcare system with our digital tools, filling in some of the gaps, for example, identifying text in the referral letters that should help patient prioritisation [38]. For example, ‘Rheumatic?’, a symptom checker questionnaire in validation and implementation in the SPIDeRR countries, could be deployed in different stages of the patient journey depending on the profile of the potential healthcare users and the specifics of the healthcare system. For instance, it can help reduce the time spent seeking help by guiding the person with symptoms to consult a rheumatologist promptly and prepare a narrative for the GP. Furthermore, symptom checkers may mitigate emotional burden not by resolving wait times but by reducing uncertainty, restoring a sense of agency, and legitimising symptoms – converting an ambiguous, passive experience into a bounded and actionable one. It can also be implemented as a tool to be used directly during the GP consultation, exploiting its potential to enhance the doctor-patient communication process. Furthermore, it could become a communication tool between healthcare levels. Additionally, this tool can counteract the patients’ feelings of frustration and being misunderstood [39].

By visually representing the journey and understanding it from the patient’s perspective, as we have done, healthcare providers can make data-driven improvements to healthcare services that enhance the quality of care and improve patients’ perceptions of the care they receive, ultimately reducing inequalities. We provide an example of how to adopt a more patient-centric approach to healthcare, aiming to enhance the overall patient experience and satisfaction. This strategy can be used, for example, when developing, testing and monitoring new technologies such as AI, which the EU is currently promoting [38], [39]. In summary, navigating healthcare systems is complex for most people, especially those with MSK issues, across all countries. Our study contributes to understanding the context in which a person with MSK symptoms enters the healthcare system and navigates through it and helps inform discussions on implementing the SPIDeRR tools in European healthcare systems.

## CRediT authorship contribution statement

**Teresa Otón:** Writing – review & editing, Writing – original draft, Methodology, Investigation, Formal analysis, Data curation, Conceptualization. **Felix Muehlensiepen:** Writing – review & editing, Methodology, Investigation, Formal analysis. **Rachel Knevel:** Writing – review & editing, Supervision. **María José Villalobos-Quesada:** Writing – review & editing, Conceptualization. **Barbara Bislawska Axnäs:** Writing – review & editing, Conceptualization. **Harriet Morf:** Writing – review & editing. **Karin Stratingh:** Writing – review & editing, Investigation. **Marina Pérez:** Writing – review & editing, Visualization, Methodology, Investigation. **Estíbaliz Loza:** Investigation, Data curation, Conceptualization. **Loreto Carmona:** Writing – review & editing, Writing – original draft, Visualization, Methodology, Investigation, Formal analysis, Data curation, Conceptualization.

## Declaration of competing interests

None of the authors declares any conflict of interest with the topic of the study.

## Appendix Supplementary materials

Image, application 1

Supplementary material Supplementary material associated with this article can be found in the online version at doi;XXXX. Image, application 2
