# Supplementary material for: The journey of Europeans with musculoskeletal complaints: the creation of the SPIDeRR’s personas
Source: EULAR Rheumatol Open. 2026 May 5;2(2):100177. doi: 10.1016/j.ero.2026.100177 (PMC13425178; doi:10.1016/j.ero.2026.100177)
Supplement: Supplementary file 1 [file mmc1.docx]

**Appendix A. The SPIDeRR consortium.**

| Servicio Madrileño de Salud (SERMAS) | Isabel Castrejon | Spain |
| --- | --- | --- |
|  | Isidoro Gonzalez | Spain |
|  | Ines Perez Sancristobal | Spain |
|  | Elena Polentinos Castro | Spain |
|  | Isabel del Cura | Spain |
|  | Maria Eugenia Miranda Carus | Spain |
|  | Carlos Betancort Rodriguez | Spain |
| Stichting Reuma Nederland (RNL) | Annelies van Ravestijn | Netherlands |
|  | Marije Hulsinga | Netherlands |
|  | Mir Blijleven | Netherlands |
| Technische Universiteit Delft (TUD) | Marcel Reinders | Netherlands |
|  | Erik van den Akker | Netherlands |
|  | Daniyal Selani | Netherlands |
|  | Inez den Hond | Netherlands |
| Thermofisher Scientific Phadia GmbH (TFS) | Linda Mathsson-Alm | Sweden |
|  | Maresa Grunhuber | Germany |
|  | Sascha Swiniarski | Germany |
| Medizinische Hochschule Brandenburg Campus Neuruppin GmbH (MHB) | Felix Muehlensiepen | Germany |
| Instituto de Salud Musculoesquelética, SL (InMusc) | Loreto Carmona | Spain |
|  | Estíbaliz Loza Santamaría | Spain |
|  | María Jesús García de Yébenes | Spain |
|  | Teresa Otón Sánchez | Spain |
| Idryma Iatroviologikon Ereunon Akademias Athinion (IIE) | Aggelos Banos | Greece |
|  | Dimitrios Boumpas | Greece |
|  | Antonis Fanouriakis | Greece |
| IQVIA Solutions BV (IQVIA) | Louise Janssen | Netherlands |
|  | Arlette Horn | Netherlands |
|  | Everdien Derksen | Netherlands |
| Erasmus University Hospital Rotterdam (EMC) | Dieuwke Schiphof | Netherlands |
|  | Laura Struik | Netherlands |
|  | Rene Suurland | Netherlands |
|  | Sita MA Bierma-Zeinstra | Netherlands |
|  | Alessandro Chiarotto | Netherlands |
|  | Premysl Velek | Netherlands |
| University of Newcastle upon Tyne (UNEW) | Arthur Pratt | UK |
|  | Heather Cordell | UK |
|  | Joe Berry | UK |
|  | James Wason | UK |
|  | Peta Leroux | UK |
|  | Natalie Yeowart | UK |
|  | Julia Scott | UK |
|  | John Isaacs | UK |
|  | Leigh Romaniuk | UK |
|  | Karina Patasova | UK |
|  | Najib Naamane | UK |
| Academic Health Science Network North East and North Cumbria (AHSN) | Julia Newton | UK |
|  | Gareth Forbes | UK |
| Semmelweis Egyetem (SEM) | Judit Majnik | Hungary |
|  | Adam Misak | Hungary |
|  | Lilla Gunkl-Toth | Hungary |
|  | Gyorgy Nagy | Hungary |
|  | Eszter Toth | Hungary |
|  | Kinga Kohalmi | Hungary |
| Universitatsklinikum Erlangen (UKER) | Harriet Morf | Germany |
|  | Katerina Berger | Germany |
|  | Anna Holtmannspoetter | Germany |
|  | Georg Schett | Germany |
|  | Anna-Maria Liphardt | Germany |
|  | Luisa Schäfers | Germany |
| University of Marburg | Johannes Knitza | Germany |
|  | Sebastian Kuhn | Germany |
| University of Manchester | John Bowes | UK |
|  | Sebastien Viatte | UK |
| Patient Advisory Board | Peter Mihajilik | Hungary |
|  | Peter Boehm | Germany |
|  | David Black | UK |
