# Supplementary material for: The journey of Europeans with musculoskeletal complaints: the creation of the SPIDeRR’s personas
Source: EULAR Rheumatol Open. 2026 May 5;2(2):100177. doi: 10.1016/j.ero.2026.100177 (PMC13425178; doi:10.1016/j.ero.2026.100177)
Supplement: Supplementary file 2 — Supplementary material Supplementary material associated with this article can be found in the online version at doi;XXXX. [file mmc2.docx]

The Journey of Europeans with Musculoskeletal Complaints: The creation of the SPIDeRR’s personas – Appendix B

[B.1. Focus groups guide. 2](#_Toc225666892)

[B.2. Health System Characteristics survey: Main source of basic healthcare coverage per country. 3](#_Toc225666893)

[B.3. Health System Characteristics survey: Cost sharing of medicines. 3](#_Toc225666894)

[B.4. Health System Characteristics survey: Access to Primary Care. 3](#_Toc225666895)

[B.5. Health System Characteristics survey: Shared acute inpatient and outpatient care costs per country. 4](#_Toc225666896)

[B.6. Health System Characteristics survey: Primary care services. 5](#_Toc225666897)

[B.7. Health System Characteristics survey: Primary Care roles and choice of providers. 5](#_Toc225666898)

[B.8. Health System Characteristics survey: Access to uses of health systems for delivery of care in the target countries. 6](#_Toc225666899)

[B.9. Themes that arose concerning the different phases of the journey. 7](#_Toc225666900)

[B.10. Strategies proposed in the focus groups for improving healthcare. 9](#_Toc225666901)

[B.11. Attributes to create the personas. 11](#_Toc225666902)

# B.1. Focus groups guide.

| **Key question/ narrative impulse** | **Check aspects** | **Concretising questions** |
| --- | --- | --- |
| Our study investigates the journeys of people with MSK complaints in Europe.  Some of you have a rheumatic disease, and others don’t. We want to hear both.  For those who don’t have a diagnosis of RMD, imagine you start with pain that doesn’t go away in your hands or your back.  What would you do? | Knowledge of the system  Help-seeking behaviour  Uncertainty  Ignorance  Overload | Who would you ask?  How would you feel?  What would you expect about going to the doctor? |
| For the ones who experienced these symptoms, what did you do? | Help-seeking behaviour  First contact | Whom did you ask?  How did you feel?  Who was your first contact in the system?  What did your first doctor/contact do? |
| What happened until you got a diagnosis? | Pre-diagnosis  Expectance  Despair  Fear | What type of doctors/professionals did you see?  What happened next?  Please describe.  How did you feel back then?  Did you have any doubts about what was going on?  What would you change about this part? |
| For those who don’t have a diagnosis of RMD, imagine they tell you have an inflammatory disease; how would you feel?  Same for degenerative disease | Initial diagnosis  Fear  Rejection  Acceptance  Release | Would that diagnosis clarify anything?  What comes to your mind when you hear that?  How would you/ did you feel back then?  Would/Did you have any questions? |
| For those with a diagnosis of an inflammatory disease like RA or spondyloarthritis,  What was it like for you?  Same for non-inflammatory diseases like OA or fibromyalgia. What was it like for you? |  |  |
| How would you feel if the specialist returned you to your family doctor?  How would you feel if the family doctor cared for your problem instead of a rheumatologist? | Management  Anger  Frustration  Release | Were you discharged or referred to another specialist? |
| How could the care process have been improved for you? |  |  |

# B.2. Health System Characteristics survey: Main source of basic healthcare coverage per country.

| **Country** | **Main source** |
| --- | --- |
| Germany | Multiple insurance funds or companies |
| Greece | A single health insurance fund (single-payer model) |
| Hungary | A single health insurance fund (single-payer model) |
| Netherlands | Multiple insurance funds or companies |
| Spain | A national health system covering the country as a whole |
| Sweden | Local health systems that serve distinct geographic regions |
| United Kingdom | A national health system covering the country as a whole |

# B.3. Health System Characteristics survey: Cost sharing of medicines.

| **Country** | **Payment scheme** |
| --- | --- |
| Germany | Co-payment of 10% of cost with a minimum of EUR 5 and a maximum of EUR 10 per item. |
| Greece | Co-insurance of 0%, 10%, or 25%, depending on the drug category. |
| Hungary | There is no co-payment for medicines provided during inpatient care and in cases of certain diseases. Elderly or disabled persons with low income receive a special card that provides them with an entitlement to free medicine. Only the HUF300 (€0.75) packing fee will be paid in this case. Otherwise, the level of copayments depends on the type of drug—the rate can range from 10% to 75%. The percentage is fixed to the price of the reference pharmaceutical. |
| Netherlands | The deductible system applies to drugs covered by the scheme. In addition to the deductible system, there are copayments: generic drugs are covered 100%, and non-generics are generally covered up to the price of the generic medicine. |
| Spain | There are different levels of contribution to co-payment of medicinal products and medical devices, based on a complex scheme, from 0 to 90%, based on the place where the medicine is dispensed, type of drug, disease, income, pensioners, social integration benefits, unemployment status, or treatments derived from an accident at work or an occupational disease. |
| Sweden | There is a deductible of SEK 1300 (€ 130) beyond which the co-insurance rate applies. The deductible diminishes stepwise as spending increases (50%, 25%, 10%). Patient cost-sharing is capped at SEK 2600 (€ 260). |
| UK | England: Co-payment of GBP £8.40 (roughly 12 €) per prescription applies. But it is possible to pay a flat fee of GBP £104 (roughly 150 €) for an unlimited number of prescriptions over a year. This flat fee can be paid in monthly instalments. Too. In addition. Many people (e.g. aged 65 or older or 15 or younger and those on benefits) are exempted. Scotland: no co-payment. Wales: no co-payment. Northern Ireland: no co-payment |

# B.4. Health System Characteristics survey: Access to Primary Care.

| **Country** | **Cost** | **Comments** |
| --- | --- | --- |
| Germany | Receive free services at the point of care | Information relates to Statutory Health Insurance. |
| Greece | Pay only user fees or co-payments (where applicable) | Only in private contracts with EOPYY physicians. In public centres no user fees or co-payments are applied |
| Hungary | No co-payment |  |
| Netherlands | Receive free services at the point of care |  |
| Spain | Receive free services at the point of care |  |
| Sweden | Pay only user fees or co-payments (where applicable) |  |
| UK | Receive free services at the point of care |  |

# B.5. Health System Characteristics survey: Shared acute inpatient and outpatient care costs per country.

| **Country** | **Inpatient** | **Outpatient** | **Lab tests** | **Imaging tests** |
| --- | --- | --- | --- | --- |
| Germany | Co-payment of EUR 10 /day. Limited to 28 days/year. | Free at the point of care for patients with statutory health insurance and selected PHI contracts. | Free at the point of care | Free at the point of care |
| Greece | Patients treated in public hospitals are typically covered without cost sharing. Cost sharing (about 30% of the relevant DRG tariff) and potential extra billing exist for patients treated in private hospitals. | Free at the point of care for public providers. | Public provider cost-sharing is typically covered without cost-sharing, and contracted private providers are covered with a low cost-sharing level. | Public provider cost-sharing is typically covered without cost-sharing, and contracted private providers are covered with a low cost-sharing level. |
| Hungary | Free at the point of care | No co-payment. | No co-payment. | No co-payment. |
| Netherlands | Deductible (the patient pays the first EUR 385 each year) | GP and district nursing care are free. GP visits have no cost, but additional care like lab tests applies to the annual €385 deductible. Physiotherapy is generally not covered except for specific chronic conditions, where patients pay for the first 21 sessions annually. Subsequent years' treatments are covered after paying for 21 sessions out-of-pocket or through supplementary insurance. | Deductible (the patient pays the first EUR 385 each year) | Deductible (the patient pays the first EUR 385 each year) |
| Spain | Free at the point of care. | Free at the point of care | Free at the point of care. | Free at the point of care. |
| Sweden | A co-payment of 120 SEK (12€)/ day is required, and in some regions, it is limited to a certain number of days. | It may vary between regions, but there is a co-payment of SEK 100-300 (€10-30) in most regions, up to a cap of SEK 1300 (€130)/12 months. | Free at the point of care. | Free at the point of care. |
| UK | Free at the point of care. | Free at the point of care | Free at the point of care. | Free at the point of care. |

# B.6. Health System Characteristics survey: Primary care services.

| **Country** | **Type of practice** |
| --- | --- |
| Germany | All forms of supply mentioned exist. Predominantly solo practice with a clear trend towards multi-specialty group practice |
| Greece | Multi-specialty group practice (a practice that is run by two or more physicians or healthcare professionals who have different specialisations) |
| Hungary | Group practice with shared patients (two or more physicians or healthcare professionals who share a common pool of patients) |
| Netherlands | Group practice with shared patients (two or more physicians or healthcare professionals who share a common pool of patients) |
| Spain | Group practice with shared patients (two or more physicians or healthcare professionals who share a common pool of patients) |
| Sweden | Group practice with shared patients (two or more physicians or healthcare professionals who share a common pool of patients) |
| United Kingdom | Group practice with shared patients (two or more physicians or healthcare professionals who share a common pool of patients) |

# B.7. Health System Characteristics survey: Primary Care roles and choice of providers.

| **Country** | **Are people registered with a GP or practice?** | **Do GPs control access to specialist care?** | **Are patients usually free to choose providers of outpatient specialist services in the community?** |
| --- | --- | --- | --- |
| Germany | No | There is no need and no incentive to obtain a GP referral | Patients do not face any incentives to choose one provider over another |
| Greece | Yes. The majority (>50%) | There is no need and no incentive to obtain a GP referral | Patients can choose any physician providing outpatient specialist services but have financial incentives to choose certain providers |
| Hungary | Yes. (almost) the whole population (99%) | GP referral is compulsory only to access certain types of specialist care. No referral is needed for dermatology, gynaecology, urology, psychiatry, and addictionology; ophthalmology, surgery, traumatology, oncology, and emergency care. | Patients are free to register with the primary care doctor of their choice; there is no incentive. |
| Netherlands | There is no incentive and no obligation to register with a GP (or practice) | GP referral is compulsory to access most types of specialist care (except in case of emergency) | Patients can choose any physician providing outpatient specialist services, but depending on their insurance, they could have financial incentives to choose certain providers |
| Spain | Yes. (almost) the whole population (99%) | GP referral is compulsory to access most types of specialist care (except in case of emergency) | The patient’s choice is limited |
| Sweden | Yes. the majority (>50%) | Patients have financial incentives to obtain a GP’s referral (e.g. reduced co-payments), but direct access is always possible | Patients do not face any incentives to choose one provider over another |
| United Kingdom | Yes. (almost) the whole population (>99%) | GP referral is compulsory to access most types of specialist care (except in case of emergency) | Patients do not face any incentives to choose one provider over another |

# B.8. Health System Characteristics survey: Access to uses of health systems for delivery of care in the target countries.

| If yes, What for? | Germany | Greece | Hungary | Netherlands | Spain | Sweden | UK |
| --- | --- | --- | --- | --- | --- | --- | --- |
| making appointments? | No | Yes |  | Yes | Yes | Yes | Yes |
| ordering and/ or receiving results of **laboratory tests**? | Yes | Yes |  | Yes | Yes | Yes | Yes |
| issuing drug prescriptions? | Yes | Yes |  | Yes | Yes | Yes | Yes |
| sending prescriptions to a **pharmacy**? | Yes | Yes |  | Yes | Yes | Yes | Yes |
| recording of **consultations**? | Yes | Yes |  | Yes | Yes | Yes | Yes |
| sending **referral letters** to medical specialists? | Yes | Yes |  | Yes | Yes | Yes | Yes |
| ordering and/or receiving **diagnostic test** results? | Yes | Yes |  | Yes | Yes | Yes | Yes |
| **storing** records on laboratory tests, filled drug prescriptions, consultations, referrals, and diagnostic tests? | Yes | Yes |  | Yes | Yes | Yes | Yes |
| storing records on **vaccinations**? | Yes |  |  | Yes | Yes |  | Yes |
| storing records on allergies and intolerances? | Yes |  |  | Yes | Yes |  | Yes |
| storing records on **medical procedures**? | Yes |  |  | Yes | Yes |  | Yes |
| storing records on **medical devices** in use? | Yes |  |  |  |  |  | Yes |
| receiving **alerts** or prompts about a potential drug dose or interaction problem? | No | Yes |  | Yes | Yes | Yes | Yes |
| receiving **alerts** or results about a consultation from **specialist**/inpatient care that needs to be followed up by GP? | No |  |  | Yes |  |  | Yes |
| receiving **alerts** about a **routine follow** of care (e.g., screening notification) or medical history of the patient (e.g., allergy, renal insufficiency) that needs to be signalled to GP? | No |  |  | Yes | Yes |  | Yes |
| To gain **access to data** from other providers to follow up on previous or inform current episodes of care across settings? | Yes |  |  | No | Yes |  | Yes |

# B.9. Themes that arose concerning the different phases of the journey.

| **Phase** | **Themes** | **Verbatim** |
| --- | --- | --- |
| Awareness and help seeking | **Delayed help-seeking** | "*I don't complain much, or I don't go to the doctor very much, only when I'm feeling like really bad*." (Female, 30-50, without RMD) |
|  | **Denial and normalisation** | "*I thought, right. I thought it's just that. Yeah. Back pain is so common*." (Female, 30-50, with RMD) |
|  | **Informal support** | "*Also, my sister is a doctor, so if I feel really bad before going to the doctor, many times I ask her or whatnot*." (Female, 30-50, without RMD) |
|  | **Self-management** | “*I would probably wait and see if it goes away. Maybe if I change something. Like to stop doing things, or start doing things… And then if it doesn't go, then I will seek a medical professional*” (Male, <30, without RMD)  "*My foot started swelling up over a weekend. So, it was a Saturday, and I knew I wouldn't be able to see my local doctor until the Monday. So, I took painkillers and put ibuprofen gel on the swelling*." (Male, 30-50, with RMD) |
|  | Misinterpretation of symptoms | "*And I haven't ever thought… maybe I was told that this is a degenerative process, but I realized this only when I just found a book about arthritis*." (Male, 30-50, with RMD) |
|  | **Alternative care** | "*He prescribed, you know, acupuncture? So, I did that, and you know, in acupuncture, you lie down for half an hour, and that was kind of nice*." (Female, 30-50, with RMD) |
| First encounter | **Primary care as a gateway** | "*I just went to my family doctor. She sent me for an x-ray to start with..."* (Female, 30-50, without RMD) |
|  | Role of GP | *“It really is difficult to access. You can refer to physiotherapy, you can self-refer, but, still, that actually takes an extremely long time."* (Female, >50, without RMD) |
|  | **Lack of immediate access** | *"I mean, I think, like […] has said, it's an extremely slow process in the UK, although we have the NHS.*" (Male, >50, without RMD) |
|  | **Difficulty in communicating symptoms** | "*But I always knew that there was something wrong, and I remember telling a friend of mine who sort of thought I was an idiot, that something is wrong with me, and it's as if, you know, imagine your right arm is missing or something, and therefore you're handicapped.*" (Female, 30-50, with RMD) |
|  | **Misdiagnosis and frustration** | "*For me, it started very early, because we had a farm at home, and I had to help, and I couldn't do the work. And so, we went to a specialist who told my parents I'm only too lazy for working...*" (Female, >50, with an RMD)  "*Because my blood tests are abnormal, they should have taken me seriously!!* (Female, <30, with RMD) |
|  | Emotional labour | "*I am 57 years old, and when you're my age, they usually get a lot of other explanations for why you have why you're in pain and stuff…* " (Female, >50, with RMD)  “*Especially if you're overweight, if I may say. They always say, “you're overweight, lose weight*!” (Female, >50, with RMD) |
|  | **Education and awareness** | "I want doctors in their studies to learn about autoimmune diseases and the way they present." (Female, 30-50, with RMD) |
|  | Anxiety and fear | "W*hen my first symptoms happened, which was very frightening because all of my fingers were painful, I booked immediately a time point to a rheumatologist because I was very afraid*." (Female, 30-50, with RMD)  "*I mean, I think, if I would find that it might be like an autoimmune disease, I would definitely be concerned because I would think that's maybe something I'm going to have for life*." (Female, <30, without RMD) |
|  | **Empathy and understanding** | "The thing I also heard from you is that I don't think I had it as much because I didn't have an idea what I have. But when doctors are condescending, and they say you're lying, you're hysteric; it's not true." (Female, 30-50, with RMD) |
|  | Persistence and resilience | *“And I try to do everything, every activity.*" (Male, 30-50, with RMD) |
| Management outside rheumatology | **Broken communication** | "*I ended up somewhere between a rheumatologist and the orthopaedic surgeon that we were discussing about what was going on with me, and my opinion, was not really important.*" (Female, 30-50, with RMD) |
|  | Frustration | "*I was so frustrated. And still to this day, it is a part-time job to do health management, you know, because I have so many things now. And this frustration; I've become used to it, but the frustration of not being believed is horrible*." (Female, 30-50, with RMD) |
|  | Despair | *"It was horrible at that time."* [about being misunderstood and not believed by medical professionals] (Female, >50, with an RMD)  “*I had started to believe that it was actually in my mind, because no one was taking me seriously”* (Female, <30, with an RMD) |
| Rheumatology referral | Access to specialist | "*When my first symptoms happened (…), which was very frightening (…) all of my fingers were painful, (…) I booked immediately a time point to a rheumatologist because I was very afraid*." (Female, 30-50, with RMD)  “*The state one, I'm not that sure about that. I mean, if I have, like, not sure what it is called, like, you know, paper from the GP, to go to the rheumatologist*…” (Male, 30-50, without RMD) |
|  | **Waiting times** | "*So, in the UK, the GPs are the gatekeepers to seeing a specialist. The first concern I always have when I try to contact my local GP is there's no guarantee as to who I will see and when I will be able to see them*.” (Male, 30-50, with RMD)  "*What I miss in this kind of treatment is more continuous monitoring from the doctor, because sometimes it takes months or years and the visit to the doctor, it's every, I don't know, two, three months or even six months*." (Male, 30-50, without RMD) |
|  | System deficiencies | "*And to get anything done, you really need to go privately initially to find out what's going on a bit better*." (Female, >50, without RMD) |
|  | **Self-referral** | "*Yeah, yeah. So here in Greece, basically, you don't really need a referral from your primary care doctor to go to any other specialist. So, you can just go, but you have to pay; of course, it's not public to do that; it's not free*." (Female, <30, with RMD) |
| Management in Rheumatology | Follow-up care | “*Yeah, that that will do for me*.” (Male, 30-50, with RMD)  “*I think that's pretty normal. So, the specialist will do maybe some extra diagnosis, tells you so okay this will be the treatment plan and the follow up is by a GP, or maybe another time, once or twice by the specialist and then afterwards by the GP, I think that's pretty normal in the Netherlands. So…”* (Male, 30-50, without RMD) |
|  | Multidisciplinary care | "*I think I was thinking more along the lines of, you know, some people have more than one condition, and they only deal with these in isolation."* (Female, 30-50, without RMD) |
|  | Impact on identity | "*I was going to the campsite with my family and travelling by car with the swimming pool, with all those swimming things, with all the air in it put in the back of my car for these like 50 meters away. And I'm just going to do it pretending that nothing is actually, burdening me*." (Female, 30-50, with RMD) |
|  | Hope and relief | "*And I believed this, and I thought that I'm in the right hands, and everybody thought that I'm in the right hands.*" (Male, 30-50, with RMD) |
|  | Empowerment through knowledge | "*You know, I could at least, you know, Google it. I could at least read up on it, and then I could make myself familiar*." (Female, >50, with RMD) |
| Return to PC | **Continuity of care** | "*After you've got like a treatment plan and, he or she did, the diagnosis, then I'm fine*." (Male, 30-50, without RMD) |
|  | **Specialist overload** | "*I don't know about the other countries, right? But in Hungary, we have the private sector and we have the state provided health care system. Right. So, I guess, a private rheumatologist wouldn't send me away because he would listen to me, I paid for it, it's private*." (Male, 30-50, without RMD) |
|  | **Patient preferences and trust in GP** | "Y*eah, I'm completely happy to be managed by my local GP, on this occasion with this particular condition at the moment*" (Male, 30-50, with RMD)  “*So, it depends when you last saw the consultant and if you still got that relationship at the moment.*" (Female, >50, without RMD) |
|  | **Resource allocation** | "*I mean, and yeah, I feel like, in general, that's kind of the tendency to, yeah, to like reach out to the specialist when it's needed, but if you can move it back to the GP to, yeah, to keep it cheaper and maybe it's not necessary or so.*" (Female, <30, without RMD) |

Abbreviations: PC, Primary care; GP, general practitioner; RMD, rheumatic and musculoskeletal disease

# B.10. Themes that arose concerning contextual factors.

| **Themes** | | **Verbatim** |
| --- | --- | --- |
| **Cross-country variations** | “*Yeah. I don't know about the other countries, right? But in Hungary, we have the private sector, and we have the state-provided health care system. Right. So, I guess a private rheumatologist wouldn't send me away because he would listen to me; I paid for it; it's private*.” (Male, 30-50, with RMD)  “*We visited some doctors in Germany, but they didn't tell us anything. They told us to have a special treatment. Then we went to Greece, and she visited the doctor who specialized in this, and he gave her the treatment. But then, in Germany, she was always going to the GP to get just the prescription for the treatment.*” (Male, 30-50, without RMD) |  |
| **Cost and sustainability** | "*I mean and yeah, I feel like in general that's kind of the tendency to yeah, to like reach out to the specialist when it's needed, but if you can move it back to the GP to, yeah, to keep it cheaper*." (Female, <30, without RMD) |  |
| **Communication and transparency** | "*I think it will totally depend on the explanation of the GP*." (Male, 30-50, without RMD) |  |
| **Financial constraints** | "*Yeah, yeah. So here in Greece, basically, you don't really need a referral from your primary care doctor to go to any other specialist. So, you can just go, but you have to pay, of course, it's not public to do that, it's not free*." (Female, <30, with RMD) |  |
| **Communication and coordination** | "F*or me also, the two main issues, access and communication. Access that I can access a specialist if it's necessary in time and not have to wait a long, long time to go*." (Male, >50, with RMD) |  |
| **Geographical barriers** | "*Each region, each hospital works in its own way and buys its own medication and so*." (Female, >50, with RMD) |  |
| **Health system prioritisation** | “*If you wait on the National Health Service, it could be a very long time, so it is best to make an appointment sort of private. And now if I have a flare up of that, I go straight to the physiotherapist and bypass the doctor.*” (Male, >50, non-inflammatory RMD)  “*And in a way, when I researched the condition, I feel at this stage I'd be wasting the rheumatologist time. Now, in time, if things get worse, things may change. But, at this point, I wouldn't be… I wouldn't be seeking… I'm quite vocal and quite involved in the system, so I know how it works, in the United Kingdom*.” (Male, 30-50, with RMD) |  |
| **Complexity of symptoms** | "*The symptoms were not really specific.*" (Female, 30-50, with RMD)  "*And, a lot of people have that too, because they do sports too much, I don't, but. So, you know, whatever I had, it was everybody else has it.*" (Female, 30-50, with RMD) |  |
| **Patient advocacy and knowledge** | “So, *I would be lost a little bit and afraid when anything happens who will be able to see me and my problem and really take care of it*.” (Female, 30-50, with RMD) |  |

# B.11. Strategies proposed in the focus groups for improving healthcare.

| **Strategies** | Description | Verbatim |
| --- | --- | --- |
| **Empowering patients through self-management** | Providing patients with educational resources, apps, and online tools to help them understand their condition and actively monitor symptoms can empower them to play a more proactive role in managing their health.  Enabling peer support and community-building among patients with similar conditions, such as through group meetings or online forums, allows them to learn from each other's experiences. | "M*aybe like... my problem then was I went to a good physiotherapist and was it was kind of a muscle unbalance so it's getting better to be like, schooled, maybe like educated in terms of what exercises can I do what helps me? What can I do by myself? Like something like this would be would be beneficial, but I but I would’ve wished for my, yeah, journey or my experience and not always the same answer that you get if you go into different doctors, you're getting five times the same question within five minutes and you're like at one point getting like, okay, so are they willing to help me or not? That's something that I would like wish.*" (Male, <30, without RMD)  "*After you've got like a treatment plan and, he or she did, the diagnosis, then I'm fine*." (Female, 30-50, with RMD)  "I'm all for sort of self-help and trying to monitor your own condition." (Male, >50, without RMD) |
| **Enhancing coordination between primary care and specialists** | Improving communication between GPs and rheumatologists is crucial for streamlining the patient journey. Clear explanations from specialists on the rationale for referring patients back to primary care, including the treatment plan and when further specialist input may be needed, can help manage patient expectations.  Establishing multidisciplinary teams and hubs that take a holistic, bespoke approach to managing patients with multiple conditions could empower patients by helping them better understand their overall situation. | "S*o, okay, I'm able to inform all my doctors. I have to go to a gastroenterologist, for instance, and I can inform him about the things my rheumatologist examined and had done with me, but they didn't communicate with each other. If they're good, they communicate to my general practitioner. But, if my rheumatologist sends information to my general practitioner, I don't think that he will send this information also to my gastroenterologist, for instance. So, I have to do this, and if someone took blood from me and the other also wants to take blood, I think it’s probably not necessary to make it double. Also, it, has more costs, more expensive to do it twice. And so, I think that is also very crucial to improve the communication not only with me but also between the specialists, between all the doctors*." (Male, >50, with RMD) |
| **Leveraging technology for monitoring and communication** | Apps and online platforms can facilitate communication between patients and their care team, allowing for regular check-ins and monitoring without the need for in-person visits.  Tailored exercise programs and app reminders can supplement physiotherapy and help patients adhere to treatment plans. | "*Greater access to remote video appointments would make it easier to access, especially if movement causes you pain. If you can fit it into your schedule, it would make it easier to get started on the journey*." (Male, <30, without RMD)  "*So, like self-diagnosing tools or apps to support the patient in this patient journey*." (Male, 30-50, without RMD) |
| **Expanding access to specialist** | Exploring alternative models, such as allowing patients to access rheumatologists without a GP referral directly, as in some countries, could reduce delays in receiving a diagnosis and starting treatment.  Increasing the number of rheumatologists and clinicians in the healthcare system is important for long-term sustainability, as self-management and GP follow-up cannot fully replace the need for specialist input. | “*So, they give you like a receipt to go there. If you're not asking, they won’t give you anything. So, you have to ask for a specific ask for that. That's something that I would wish that it would be easier to get access to*" (Male, <30, without RMD) |
| **Providing emotional support** | Recognising the emotional impact of receiving a chronic MSK diagnosis and providing counselling or support groups alongside medical treatment can help patients cope with the uncertainty and lifestyle changes that may come with their condition. | “*But if we would have a very ideal world, what I would recommend to also give psychological help or psychologist help to these people also automatically because to learn and stress handling the relaxation, things like that, also help everyone to cope with the disease*." (Female, 30-50, with RMD)  "I *think it's a good point, that it depends on if you're new to the disease or that you're already getting treatments*." (Female, 30-50, without RMD) |

# B.12. Attributes to create the personas.

| **Category** | **Attributes (Level)** | | | | | | | | | | | |
| --- | --- | --- | --- | --- | --- | --- | --- | --- | --- | --- | --- | --- |
| Age | <25  25 - 35  36 - 40 | 41 - 45  46 - 50  51 - 55 | | | | | 56 - 60  61 - 65  66 - 70 | | | | | 71 - 75  76 - 80  >80 |
| Gender | Man | | | | | | Woman | | | | | |
| Socio-economic status | Low | | Middle | | | | | | | | High | |
| Country | Germany | Greece | Hungary | | | Netherlands | | Sweden | | | Spain | UK |
| Health issues | Low back pain > 3 months | Pain in hands | | | Generalised MSK pain | | | | Episodes of acute pain in feet | | | MSK pain + depression |
| Personality | Not very calm and rational  Positive  Technologically savvy  Doesn't want to bother  Doesn’t complain  Understanding  Likes to create community  Not very resourceful  Stubborn  Cares about sustainability  Likes to share their experience with others  Googles everything | | | Fearful  Likes to keep to themselves  Persistent  Problem-solver  Doesn’t like taking painkillers  Complains a lot  Often in denial  Prone to depression  Doesn’t like visiting doctors  Emotional  Self-sufficient  Prone to catastrophic thinking | | | | | | Likes competent and experienced people  Very good at communicating  Sceptical about internet information  Empathetic  Complains a lot  Easily overwhelmed  Trust advice from doctors  Lazy  Assertive  Likes it when things happen the way they should  Very rational  Spiritual | | |
| Motivations / assets | Loves going to the gym  Gives importance to health in their future  Wants to take care of their children  When they don’t get an answer, they research themselves | | | Sense of achievement when managing things on their own  Great at following instructions  Being able to play football with friends is crucial | | | | | | Prefers to make lifestyle changes than take pills  Learns from past experiences | | |
| Frustrations / barriers | Don't feel seen by other people  Past experiences have made them sceptical  They can’t lift their kid because it hurts  Their life doesn’t feel the same  Went through a divorce while they were in pain | | | Hates to repeat things; if they said it once, they don’t want to have to say it again  Their poor financial situation makes everything worse  Don’t go to the doctor unless they are feeling terrible  Doesn’t think their pain is unordinary; it is not important, and some people have it worse | | | | | | It angers them when they are not being taken seriously  It depresses them when they are not being taken seriously  They don’t have time to take care of themselves  They tend to normalize things when they happen  This one is in use! | | |
